# Topology and enzymatic properties of a canonical *Polycomb* repressive complex 1 isoform

**DOI:** 10.1002/1873-3468.13442

**Published:** 2019-05-29

**Authors:** Matteo Colombo, Ombeline Pessey, Marco Marcia

**Affiliations:** ^1^ European Molecular Biology Laboratory, Grenoble Outstation Grenoble France

**Keywords:** cell fate, chromatin, chromatin remodelling complexes, gene silencing, *Polycomb*, ubiquitin‐ligase

## Abstract

*Polycomb* repressive complex 1 (PRC1) catalyses monoubiquitination of histone H2A on Lys119, promoting gene silencing. Cells at different developmental stages and in different tissues express different PRC1 isoforms. The topology, subunit composition, structural architecture and molecular mechanism of most of these isoforms are still poorly characterized. Here, we have purified a PRC1 isoform comprising subunits RING1B, PCGF2, CBX2 and PHC2, two stable subcomplexes (RING1B‐PCGF2 and RING1B‐PHC2) and the catalytic subunit RING1B in isolation. By crosslinking mass spectrometry, we identified novel interactions between RING1B and the three non‐catalytic subunits. Biochemical, biophysical, and enzymatic data suggest that CBX2 and PHC2 play a structural role, whereas PCGF2 also modulates catalysis. Our data offer insights into the molecular architecture of PRC1 and its histone ubiquitination activity.

## Abbreviations

**ACN**, acetonitrile

**cPRC1**, canonical PRC1

**DSS**, di‐succinimidyl‐suberate

**H3K27me3**, trimethylated histone H3 on Lys27

**PRC1**,* Polycomb* repressive complex 1

**PRC2**,* Polycomb* repressive complex 2

**SAXS**, small angle X‐ray scattering

**SEC**, size exclusion chromatography

**vPRC1**, variant PRC1

**XL‐MS**, crosslinking mass spectrometry

*Polycomb* repressive complex 1 (PRC1) is an E3‐ubiquitin ligase that catalyses the monoubiquitination of Lys119 on histone H2A (H2AK119Ub) 1, 2. PRC1 was first discovered in *Drosophila* as responsible for *Hox* genes silencing 2. In humans, PRC1 is involved in embryonic development, stem cell maintenance and cell fate decision 3.

**Figure 1:**
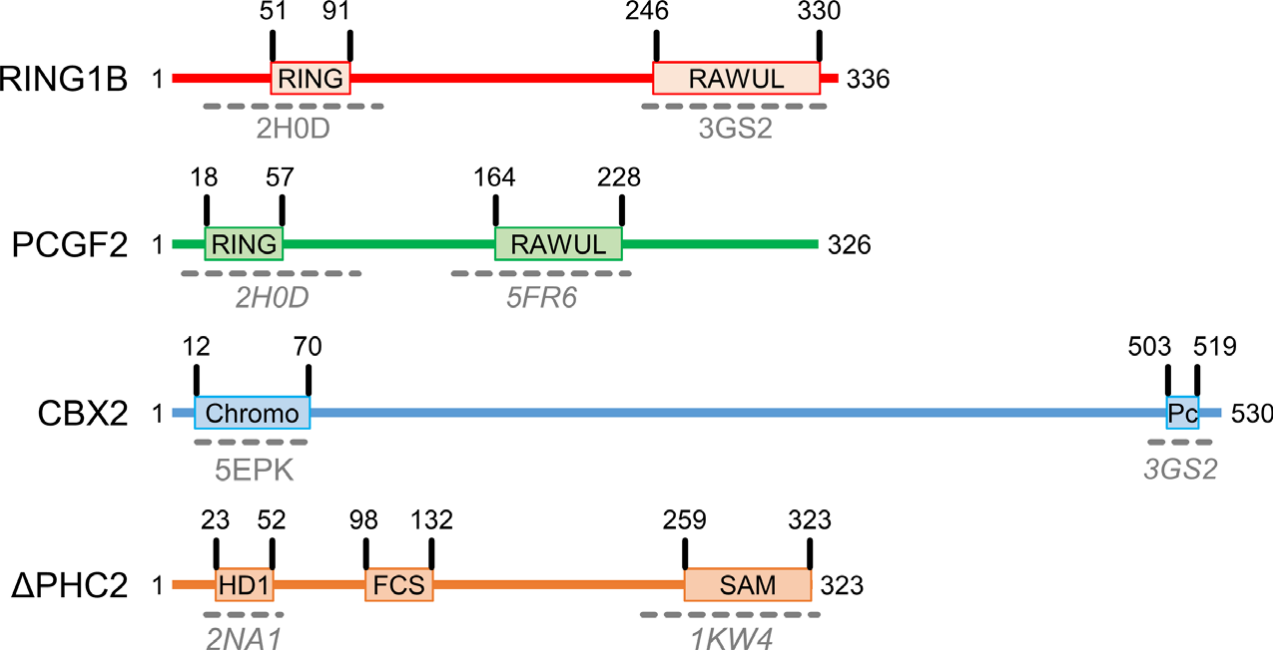
Schematic view of the subunits forming the ΔPRC1.2 complex. The figure is drawn to scale based on the length of each subunit. Regions of known structure are indicated by dashed grey lines below each domain. PDB identification numbers are indicated in grey. Where structures are available for homologous domains but not for the specific subunit used in this work, the PDB id number is indicated in italic. Based on the available structures, 70% of the cPRC1 structure is still unknown.

Human PRC1 is classified into canonical (cPRC1) and variant (vPRC1) isoforms. Canonical PRC1 monoubiquitinates H2AK119 at genomic loci where *Polycomb* repressive complex 2 (PRC2) has deposited H3K27 trimethylation marks, while vPRC1 modifies chromatin independent of PRC2 4. Here, cPRC1 comprises different isoforms, which are expressed at various stages of cell differentiation and in different tissues 5. All cPRC1 complexes are formed by a heterodimer composed of subunits RING1B and PCGF2 (aka Mel18) or PCGF4 (aka BMI1). This heterodimer associates with one CBX subunit orthologue, which contains a chromodomain recognising the H3K27me3 mark posed by PRC2 6, 7, and with one PHC subunit orthologue, which promotes PRC1 self‐association or interaction with other protein partners through its SAM domain 8, 9, 10. The RING domain of RING1B is responsible for the E3 ligase activity and it is stimulated by the RING domain of PCGF2/4 9, 11. Instead, CBX is responsible for inducing chromatin compaction *via* a non‐enzymatic mechanism 6, 7, 12. The Pc box domain of CBX7, conserved among CBX subunits, has been crystallised in complex with the RAWUL domain of RING1B 13. Moreover, the HD1 motif of PHC2 has been crystallised in complex with the RAWUL domain of PCGF4 14. The structure of the heterodimer of the RING domains of RING1B and PCGF4 in complex with mononucleosomes is also available 15. However, the molecular mechanism of RING1B stimulation is not well understood, partly because there is a lack of biochemical, biophysical and structural evidence on the molecular organisation and topology of PRC1 (Fig. 1). Specifically, the role of CBX and PHC subunits in complex assembly and in catalysis is unclear.

To improve understanding of the molecular properties of cPRC1, here we have purified a cPRC1 isoform, two stable subcomplexes, and the catalytic subunit RING1B in isolation. We report the topological map of this cPRC1 isoform obtained by crosslink in mass spectrometry (XL‐MS). Based on enzymatic, biochemical and SAXS data on this isoform and comparison to its subcomplexes, we discuss the role played by each non‐catalytic subunit. We attribute a structural, but not an enzymatic role to CBX and PHC. Moreover, we observe that PCGF stimulates RING1B catalysis and we suggest that such stimulation is partly due to the fact that PCGF increases the affinity of RING1B for the nucleosomes and reduces the affinity of RING1B for the E2 enzyme thus increasing the E2 enzyme turnover.

## Materials and methods

### Cloning, expression and purification

We purchased cDNA I.M.A.G.E. clones of each PRC1 subunits from Source BioScience (Nottingham, UK). The I.M.A.G.E. clones numbers are: RING1B = 4285715, PCGF2 = 3841545, PCGF4 = 4138748, PHC2 = 40146661 and CBX2 = 100062386. The formation of PRC1.2, PRC1.4, ΔPRC1.2 and their subcomplexes was carried out using the MultiBac technology 16. Coding sequences of individual subunits were cloned into acceptor (p‐ACEBAC‐1) and donor (pIDC, pIDS and pIDK) vectors of the MultiBac system by sequence‐and‐ligation‐independent cloning and fused by *in vitro* Cre–loxP recombination to yield a single plasmid with multiple expression cassettes (primers are listed in Table 1) 17. The presence of the gene encoding each subunit in the corresponding construct was verified by restriction enzyme digestion. The presence of an in‐frame insert was verified by DNA sequencing. Recombinant baculovirus was produced as previously described 18 and used to infect Sf21 insect cells at a cell density of 1.0 × 10^6^ per mL in SF900 medium. Cells were collected 72–96 h after proliferation arrest by centrifugation at 1000 ***g*** for 15 min and stored at −20 °C. Each PRC1 complex from 2 to 3 L pellet was resuspended in 200 mL lysis buffer (HEPES 50 mm pH 8, NaCl 150 mm, 1 mm DTT, 0.1% NP40, 1 mm Leupeptine and 1 mm Pepstatine) by vortexing. The mixture was sonicated on ice for 8 min at 35% intensity using a Sonics VCX‐750 Vibra Cell Sonicator (Sonics & Materials Inc., Newtown, CT, USA). The lysate was centrifuged 1 h at 38 500 ***g*** at 4 °C. The supernatant was loaded onto 7 mL pre‐equilibrated beads of strep‐tactin resin and the flow‐through was collected by gravity flow. The resin was washed with 13 CV of washing buffer (HEPES 50 mm pH 8, NaCl 150 mm, 1 mm DTT). The PRC1 complexes were eluted using 6–7 CV of elution buffer (HEPES 50 mm pH 8, NaCl 150 mm, 1 mm DTT, 5 mm desthiobiotin). PRC1 complexes were loaded onto an S200 16/600 column (GE Healthcare Europe GmbH, Velizy‐Villacoublay, France) pre‐equilibrated in washing buffer. Fractions containing the targets were pooled and concentrated using a 15 mL Amicon 50 kDa molecular weight cut off and injected onto an S200 10/300 column (GE Healthcare). Fractions containing the targets after this second size exclusion chromatographic step were pooled and used for subsequent experiments.

**Table 1:** Primers used to produce the constructs of this study.

Subunit or vector	Forward primer (5′–3′)	Reverse primer (5′–3′)
RING1B	GCGGATCCCGGTCCGAAGATGAGCGCTTGGAGCCACCCGCAGTTCGAAAAAGAAAACCTGTACTTTCAAGGTTCTCAGGCTGTGCAGACAAACG	GAGACTGCAGGCTCTAGATTCTCATTTGTGCTCCTTTGTAGGTGC
PCGF2	GATCACCCGGGATCTCGAGATGCATCGGACTACACGG	CATCTCCCGGTACCGCATGTCAAGTTAAGGGGGGCACG
PCGF4	GATCACCCGGGATCTCGAGATGCATCGAACAACGAGAATC	CATCTCCCGGTACCGCATGTCAACCAGAAGAAGTTGCTGATG
CBX2	GATCACCCGGGATCTCGAGATGCACCATCACCATCACCATCTGGAAGTGCTGTTTCAGGGCCCGGAGGAGCTGAGCAGCGTGG	CATCTCCCGGTACCGCATGTCAGTAATGCCTCAGGTTGAAGAAGC
PHC2	GCGGATCCCGGTCCGAAGATGGAGAATGAGCTGCCAGTCC	GAGACTGCAGGCTCTAGATTCCTAGGAGTCCTTGAGCATGCTG
ΔPHC2	GCGGATCCCGGTCCGAAGATGTACCCATACGATGTTCCAGATTACGCTACCTCAGGGAACGGAAACTCTGC	GAGACTGCAGGCTCTAGATTCCTAGGAGTCCTTGAGCATGCTGATGC
pACEBAC	GAATCTAGAGCCTGCAGTCTC	CTTCGGACCGGGATCCGC
pIDS	CATGCGGTACCGGGAGATG	CTCGAGATCCCGGGTGATC
pIDK	CATGCGGTACCGGGAGATG	CTCGAGATCCCGGGTGATC
pIDC	GAATCTAGAGCCTGCAGTCTC	CTTCGGACCGGGATCCGC

### Peptide mass fingerprinting mass spectrometry

ΔPRC1.2 and RING1B‐ΔPHC2 complexes were separated by SDS/PAGE following staining with Coomassie Brilliant Blue G250 [0.4% (w/v), 10% (w/v) citric acid, 8% (w/v) ammonium sulphate, 20% (v/v) methanol]. Coomassie‐stained bands were excised, chopped into small pieces and transferred to 0.5 mL Eppendorf tubes. For all following steps, buffers were exchanged by two consecutive 15 min incubation steps of the gel pieces with 200 μL of acetonitrile (ACN) whereby the ACN was removed after each step. Proteins were reduced by the addition of 200 μL of a 10 mm DTT solution in 100 mm ammonium bicarbonate (AmBic) and incubated at 56 °C for 30 min. Proteins were alkylated by the addition of 200 μL of a 55 mm iodoacetamide solution in 100 mm AmBic and incubated for 20 min in the dark. Fifty microlitre of trypsin at 1 ng·μL^−1^ were added to the gel pieces, incubated for 30 min on ice and then overnight at 37 °C. Gel pieces were sonicated for 15 min, spun down and the supernatant was transferred into a glass vial. Remaining gel pieces were washed with 50 μL of an aqueous solution of 50% ACN and 1% formic acid and sonicated for 15 min. The combined supernatants were dried in a Speedvac rotary evaporator and reconstituted in 10 μL of an aqueous solution of 0.1% (v/v) formic acid. Peptides were separated using the nanoAcquity UPLC system with a nanoAcquity trapping and analytical column, which was coupled to an LTQ Orbitrap Velos (Thermo Fisher Scientific, Waltham, MA, USA) using the Proxeon nanospray source. Full scan MS spectra with a mass range of 300–1700 *m/Z* were acquired in profile mode with a resolution of 30.000 and a filling time of 500 ms applying a limit of 10^6^ ions. The 15 most intense ions were fragmented in the LTQ using a normalised collision energy of 40%. 3 × 10^4^ ions were selected within 100 ms and their fragmentation was achieved upon accumulation of selected precursor ions. MS/MS data were acquired in centroid mode of multiple charged (2+, 3+, 4+) precursor ions. The dynamic exclusion list was restricted to 500 entries with a maximum retention period of 30 s and relative mass window of 10 p.p.m. In order to improve the mass accuracy, a lock mass correction using a background ion (*m/Z* 445.12003) was applied. Acquired data were processed using isobarquant 19 and mascot (v2.2.07) (Matrix Science, Boston, MA, USA) using a reversed Uniprot *Homo sapiens* database (UP000005640) including common contaminants. The following modifications were taken into account: carbamidomethyl (C) (fixed modification), acetyl (N‐term) and oxidation (M) (variable modifications). The mass error tolerance for full scan MS spectra was set to 10 p.p.m. and for MS/MS spectra to 0.02 Da. A maximum of two missed cleavages were allowed. A minimum of two unique peptides with a peptide length of at least seven amino acids and a false discovery rate below 0.01 were required on the peptide and protein level to consider the result significant.

### Nucleosome production

About 50 μL of TOP10 competent cells was transformed with 10 ng of pST55 plasmid containing 16 copies of 147 bp 601 Widom DNA 20. Widom 601 DNA was then purified as described 21. Nucleosomes were reconstituted as described 22 (Fig. S3  and Table 4). Fluorescently labelled nucleosomes were produced by cloning a cysteine‐free variant of wild type nucleosomes (H3‐C110S), introducing a cysteine residue in position 10 of H2A (H3‐C110S/H2A‐T10C double mutant) and finally labelling the double mutant with Cy5‐maleimide, as described 15.

### Crosslinking mass spectrometry

About 50 μg of purified ΔPRC1.2, RING1B‐PCGF2 and RING1B‐ΔPHC2 complexes were individually crosslinked by addition of 5 μL at 50 mm of an iso‐stoichiometric mixture of H12/D12 isotope‐coded di‐succinimidyl‐suberate (DSS) and incubated at 37 °C for 30 min. Reaction was quenched by addition of ammonium bicarbonate to a final concentration of 50 mm for 10 min at 37 °C. Crosslinked proteins were denatured using urea and Rapigest at a final concentration of 4 m and 0.05% (w/v), respectively. Samples were reduced using 10 mm DTT (30 min at 37 °C), and cysteines were carbamidomethylated with 15 mm iodoacetamide (30 min in the dark). Protein digestion was performed first using 1 : 100 (w/w) LysC (Wako Chemicals, Neuss, Germany) for 4 h at 37 °C and then finalised with 1 : 50 (w/w) trypsin overnight at 37 °C, after the urea concentration was diluted to 1.5 m. Samples were then acidified with 10% (v/v) trifluoroacetic acid (TFA) and desalted using OASIS^®^ HLB μElution Plate Crosslinked peptides were desalted and reconstituted with SEC buffer [30% (v/v) ACN in 0.1% (v/v) TFA] and fractionated using a Superdex Peptide PC 3.2/30 column (GE Healthcare). Collected fractions were analysed by liquid chromatography‐coupled tandem mass spectrometry (MS/MS) using a nanoAcquity UPLC system connected online to LTQ‐Orbitrap Velos Pro instrument. To assign the fragment ion spectra, raw files were converted to centroid mzXML using a raw converter and then searched using xquest 23 against a FASTA database containing the sequences of the crosslinked proteins. Posterior probabilities were calculated using xprophet, and results were filtered using the following parameters: FDR = 0.05, min delta score = 0.95, MS1 tolerance window of 4–7 p.p.m., ld score > 25. Data are represented with circos (http://circos.ca/).

### Microscale thermophoresis

Microscale thermophoresis was used to determine the *K*_d_ between UbcH5c E2 enzyme and RING1B/RING1B‐PCGF2. UbcH5c was expressed in *Escherichia coli* Rosetta (DE3) overnight at 18 °C. Cells were harvested by centrifugation at 5000 ***g*** for 15 min at 4 °C. Two litre cell culture was resuspended in TrisHCl 50 mm pH 7.5 at 4 °C, NaCl 150 mm, 1 mm DTT, and anti‐protease Complete EDTA‐free tablets (Roche, Basel, Switzerland). UbcH5c was sonicated 5 min on ice and centrifuged at 27 500 ***g*** for 1 h. The UbcH5c supernatant was incubated with 4 mL GST resin for 2 h in TrisHCl 50 mm pH 7.5 at 4 °C, NaCl 150 mm, 1 mm DTT. The resin was washed with 20 mL of washing buffer (50 mm Tris pH 7.5 at 4 °C, NaCl 150 mm, 1 mm DTT). UbcH5c was eluted with 15 mL of TrisHCl 50 mm pH 7.5 at 4 °C, NaCl 150 mm, 1 mm DTT and 10 mm glutathione. UbcH5c was incubated with 3C protease in 1 : 100 ratio to cleave GST tag and dialysed overnight to remove glutathione in 1 L TrisHCl 50 mm pH 7.5 at 4 °C, NaCl 150 mm, 1 mm DTT. Then, UbcH5c was loaded on Ni‐NTA resin to remove GST tag. We recovered the flow‐through, concentrated it and loaded it on an S200 16/600 in TrisHCl 50 mm pH 7.5 at 4 °C, NaCl 150 mm, 1 mm DTT. Fractions containing UbcH5c were pooled, concentrated and labelled with NT‐547 fluorescent dye (NanoTemper Technologies GmbH, Munich, Germany) according to the manufacturer procedure. UbcH5c enzyme, RING1B and RING1B‐PCGF2 were dialysed using mini slide‐A‐lyser tubes in HEPES 50 mm pH 8, NaCl 75 mm, DTT 1 mm, Tween 0.05% overnight at 4 °C under stirring. We prepared 10 μL serial dilutions of RING1B and RING1B‐PCGF2 in 16 PCR tubes at 2× final concentration (starting at 80 μm for RING1B and 38 μm for RING1B‐PCGF2). We added 10 μL of labelled UbcH5c enzyme at 200 nm to yield a final concentration of 100 nm. The mixture from each tube was loaded in a hydrophilic capillary and introduced in the sample holder of the Monolith Instrument NT.115 (Munich, Germany). LED power was set to 20% and MST power to 40%. *K*_d_ was calculated by fitting data points from two independent experiments with graphpad (GraphPad Software, San Diego, CA, USA).

### Size exclusion chromatography‐small angle X‐ray scattering

Size exclusion chromatography (SEC) small angle X‐ray scattering (SAXS) experiments were performed on the BM29 beamline at the European Synchrotron Radiation Facility (ESRF, Grenoble, France). An online HPLC system was attached directly to the sample‐inlet valve of the beamline sample changer. Fifty microlitre of ΔPRC1.2 at 2.9 mg·mL^−1^ and 50 μL at 3.6 mg·mL^−1^ of RING1B‐PCGF2 were manually injected on an S200 15/150 column, respectively. The column was pre‐equilibrated with buffer HEPES 50 mm pH 8, NaCl 150 mm, DTT 1 mm. Buffers were degassed and a flow rate of 0.2 mL·min^−1^ at 4 °C was used for all sample runs. Prior to each run, the column was equilibrated with 2 CV of buffer and the baseline was monitored. All data from the run were collected at a wavelength λ = 0.99 Å using a sample‐to‐detector (PILATUS 1M; Dectris AG, Baden, Switzerland) distance of 2.87 m corresponding to a *q*‐range of 0.0035–0.167 Å^−1^ where *q* is the momentum transfer (*q *= 4πλ sinθ) and 2θ the scattering angle. Approximately 900 frames with an exposure time of 1 s per frame were collected per sample run. 100 initial frames were averaged to create the reference buffer and the frames collected from each elution peak (40 frames/peak for both ΔPRC1.2 and RING1B‐PCGF2), corresponding to the scattering of an individual purified species, were also averaged and subtracted from the reference buffer using the program primus 24. Radii of gyration (*R*_g_) and pairwise distance distribution functions [*P*(*r*)] were extracted based on the Guinier approximation.

### H2A monoubiquitination activity assay

A 3×‐concentrated master mix containing Na‐HEPES 50 mm pH 7.7, 90 nm E1 enzyme (BML‐UW9410‐0050; Enzo Life Sciences, Villeurbanne, France), 1.2 μm UbcH5c, 10 mm DTT, 6 mm ATP, 30 μm ZnSO_4_, 15 mm MgCl_2_, 23 μm methylated ubiquitin (U‐501‐01M; R&D System, Minneapolis, MN, USA) was pre‐heated at 37 °C for 20 min. ΔPRC1.2, RING1B‐PCGF2, RING1B‐ΔPHC2 and RING1B were prepared at 10 concentrations (0.6, 0.9, 1.2, 1.5, 1.8, 2.1, 2.4, 3, 3.6, 4.2 μm) in final buffer HEPES 50 mm pH 8, NaCl 75 mm, DTT 1 mm. Nucleosomes were prepared at 2.1 μm in TE buffer with 150 mm NaCl. Finally, 15 μL of master mix 3×, 15 μL of each concentration of each PRC1 complex and 15 μL of nucleosomes were mixed and incubated at 37 °C for 100 min. The reaction was quenched by adding SDS buffer (5% glycerol, 2% β‐mercaptoethanol, 50 mm Tris‐HCl pH 8.0, bromophenol blue, 2% SDS) and boiling at 95 °C. All samples were loaded on pre‐casted SDS Tris‐glycine 4–20% polyacrylamide gels (Life Technologies, Carlsbad, CA, USA). For detection of Cy5‐labelled nucleosomes, gels were scanned with a ChemiDoc Imaging System (BioRad, Hercules, CA, USA), before Coomassie blue staining (Figs S5 and S6). For western blot detection (Fig. S2), gels were transferred on nitrocellulose membrane (Dutscher, Brumath, France) using a power supplier at 100 V for 1 h. Each membrane was blocked overnight with a 2% solution of BSA diluted in Tris buffer saline with Tween 20 (TBST). The membranes were washed with TBST and incubated with anti‐H2AUb antibody (1 : 600, 06‐678; Millipore, Burlington, MA, USA) and anti‐H2A (1 : 2500, 07‐146; Sigma Aldrich, Saint‐Louis, MO, USA) for 4 h at RT. The anti‐H2A antibody displays cross‐reactivity with histone H4 and it is outcompeted by the anti‐H2AUb antibody, when used simultaneously on ubiquitinated H2A 25. Membranes were washed with TBST. We incubated the membranes 2 h with the secondary antibodies anti‐mouse (1 : 5000, A11002; Thermo Fisher Scientific, for anti‐H2AUb) conjugated with Alexafluor dye 532 nm and anti‐rabbit (1 : 5000, A32731; Thermofisher, for anti‐H2A) conjugated with Alexafluor dye 488 nm. The membranes were washed and the fluorescent signal from the membranes was recorded with a Typhoon trio scanner. The bands were quantified with Quantity One (BioRad). The H2A monoubiquitination activity was calculated as the ratio between ubiquitinated and total H2A and plotted over the concentrations of PRC1 complexes. Data were analysed using graphpad (GraphPad Software).

### E2‐discharging assay (single turnover monoubiquitination assay)

E2‐discharging assays were performed as described 26, 27. Briefly, a 3×‐concentrated master mix containing Na‐HEPES 50 mm pH 7.7, 90 nm E1 enzyme, 1.2 μm E2 enzyme, 6 mm ATP, 30 μm ZnSO_4_, 15 mm MgCl_2_, and 23 μm methylated ubiquitin was pre‐heated at 37 °C for 1 h to charge the E2 enzyme with ubiquitin (Fig. S6A). The mix was then supplemented with 4.5 U·mL^−1^ apyrase (NEB # M0398S) to deplete ATP and incubated at 37 °C for another hour. RING1B and RING1B‐PCGF2 were then prepared at 3 μm in Na‐HEPES 50 mm pH 8 and NaCl 75 mm, and Cy5‐labelled nucleosomes were prepared at 0.3 μm in TE buffer with 150 mm NaCl. Apyrase‐treated master mix, Cy5‐labelled nucleosomes, and the relevant PRC1 subcomplex were then mixed in a 1 : 1 : 1 ratio in a total volume of 165 μL. The reaction was incubated at 37 °C. 15 μL were harvested at the indicated time points (see Fig. 5D and Fig. S6) and quenched by addition of 5 μL SDS‐containing gel loading buffer and boiling at 95 °C for 5 min. Samples were then analysed by SDS/PAGE and visualised with a ChemiDoc Imaging System (BioRad), before Coomassie blue staining (Fig. 5D and Fig. S6). The bands were quantified with Quantity One (BioRad). The H2A monoubiquitination activity was calculated as the ratio between ubiquitinated and total H2A. Data points were analysed using graphpad (GraphPad Software).

## Results

### Biochemical characterisation of human cPRC1 complexes

Based on reported proteomic studies 9, 28, we assembled canonical PRC1.2 (RING1B, PCGF2, CBX2 and PHC2) and PRC1.4 (RING1B, PCGF4, CBX2 and PHC2) complexes to check if their subunits can be co‐expressed as complexes in heterologous expression systems and purified. We reconstituted both complexes using the MultiBac technology and we expressed them in Sf21 insect cells. We used strep‐tagged RING1B as bait for purification. We observed that all the subunits were pulled‐down, indicating that CBX2 and PHC2 can interact with the heterodimer formed by RING1B‐PCGF2/4, in agreement with proteomic data (Fig. S1) 9, 28. However, subsequent purification steps of PRC1 complexes by gel filtration or ion exchange chromatography showed that PHC2 and CBX2 tend to induce aggregation of the complexes into higher order oligomers.

**Figure 2:**
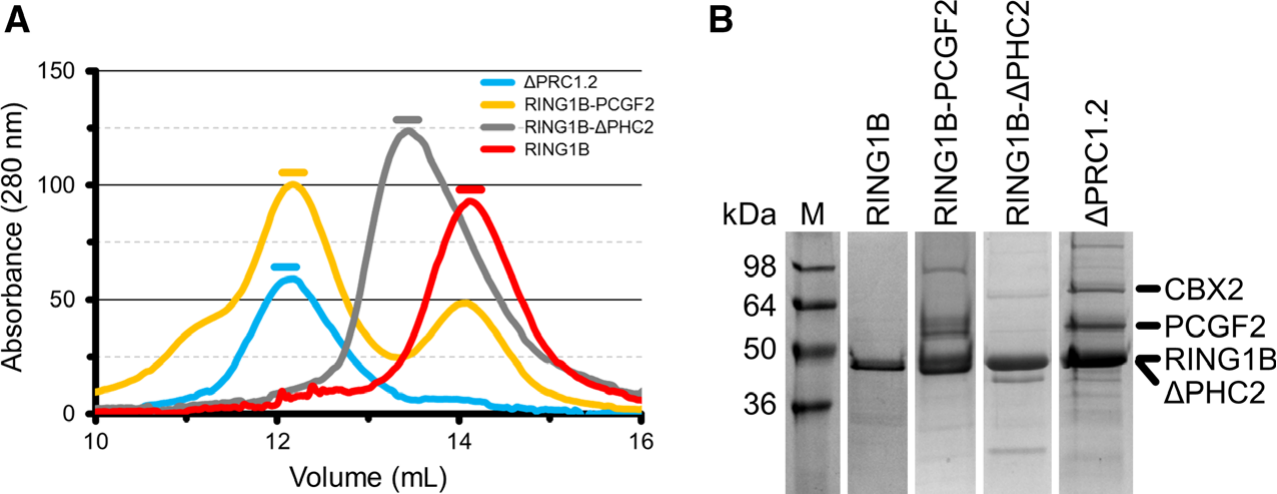
Purification of ΔPRC1.2 and of its subcomplexes. (A) SEC of ΔPRC1.2 and its subcomplexes using a Superdex 200 10/300 column. Coloured lines above each peak of absorption indicate the fractions used for downstream analysis. The RING1B‐PCGF2 sample shows an additional elution peak at *V*_e_ ~ 14.1 mL, which corresponds to excess monomeric RING1B, besides the main elution peak at *V*_e_ ~ 12.2 mL. Reinjecting the peak at 12.2 mL onto SEC (i.e. as done for SEC‐SAXS, see Fig. 3A) produces a single homogeneous peak eluting at the same retention volume. (B) SDS/PAGE of purified ΔPRC1.2 and its subcomplexes. The sizes of molecular weight markers are indicated on the left in kilodalton (kDa). Location of each subunit is indicated by a black trait on the right. Notably, ΔPHC2 and RING1B show the same electrophoretic mobility.

In human and mammalian cells, a shorter version of PHC2 (ΔPHC2), missing the first 535 amino acids, is preferentially expressed over full length PHC2 29, 30 and it associates with other PRC1 subunits 28. Moreover, the L307R mutation in the SAM domain of ΔPHC2 reduces its polymerisation propensity 10. Thus, we assembled L307R‐ΔPHC2 with RING1B, PCGF2/4 and CBX2 in MultiBac. We could purify the complex formed by RING1B, PCGF2, ΔPHC2 and CBX2 (ΔPRC1.2) in a homogeneous form (Fig. 2 and Table 2). Besides ΔPRC1.2, we could also produce and purify to homogeneity two stable subcomplexes of this isoform, namely the RING1B‐PCGF2 and RING1B–ΔPHC2 heterodimers, and the catalytic subunit RING1B in isolation (Fig. 2 and Table 3).

**Table 2:** Identification of all the subunits in the ΔPRC1.2 complex by peptide mass fingerprinting mass spectrometry.

Protein_id	top3	ssm	usm	upm	max_score	total_score	% Sequence coverage
CBX2	6.060755	26	21	19	98	1104	34.9
PCGF2	6.822288	90	28	24	99	1284	59.7
ΔPHC2	5.780417	76	43	38	156	2079	77.8
RING1B	8.062574	332	40	33	132	2532	86.4

### Full‐length PRC1 complex is more compact than RING1B‐PCGF2 heterodimer

**Figure 3:**
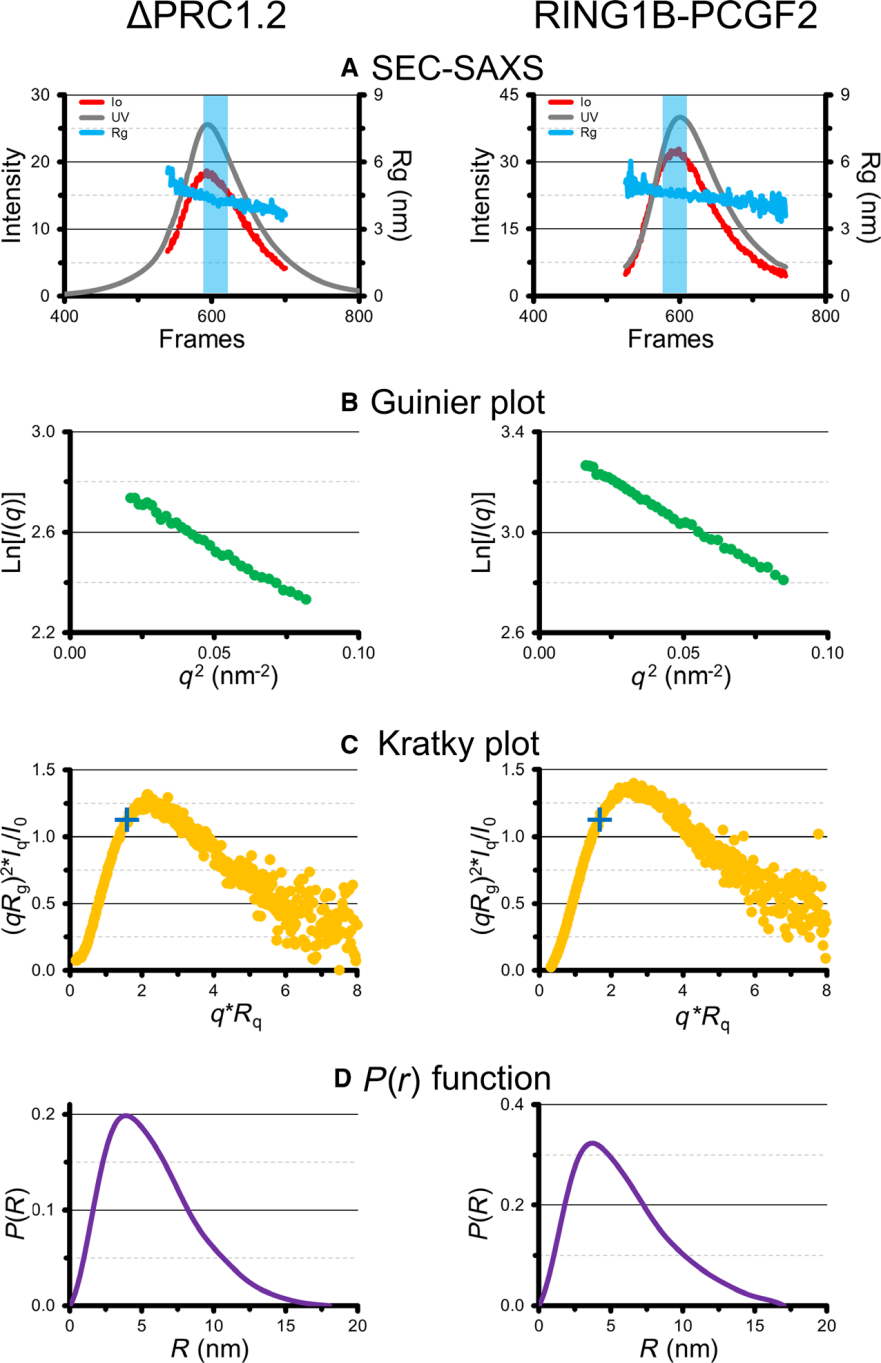
SAXS experiments of ΔPRC1.2 (left) and RING1B‐PCGF2 (right). (A) SEC and scattering profiles. The light blue rectangle highlights the frames used for SAXS data processing. (B) Guinier plots. (C) Kratky plots. The blue crosses indicate the position of the peak for an expected globular protein. (D) *P*(*r*) distribution functions.

To obtain information on the size and shape of the ΔPRC1.2 complex we performed SEC‐SAXS measurements. From the Guinier plot we calculated an *R*_g_ of 4.4 nm, while the pair distribution function [*P*(*r*)] indicates a *D*_max_ of 18 nm. The normalised Kratky plot shows a shift from the theoretical peak value expected for a globular protein, suggesting that ΔPRC1.2 displays regions of flexibility (Fig. 3), as expected from secondary structure prediction of its subunits (Fig. 1).

To understand how ΔPHC2 and CBX2 affect the shape and flexibility of ΔPRC1.2, we collected an SEC‐SAXS dataset for the RING1B‐PCGF2 subcomplex. Interestingly, data analysis shows that RING1B‐PCGF2 has *R*_g_ of 4.7 nm and a *D*_max_ of 17 nm. Thus, the heterodimer RING1B‐PCGF2 has similar *R*_g_ and *D*_max_ compared to ΔPRC1.2, suggesting that RING1B‐PCGF2 adopts a more relaxed conformation in the absence of CBX2 and ΔPHC2 (Fig. 3).

### The FCS zinc finger domain of ΔPHC2 interacts with RING domain of RING1B

**Figure 4:**
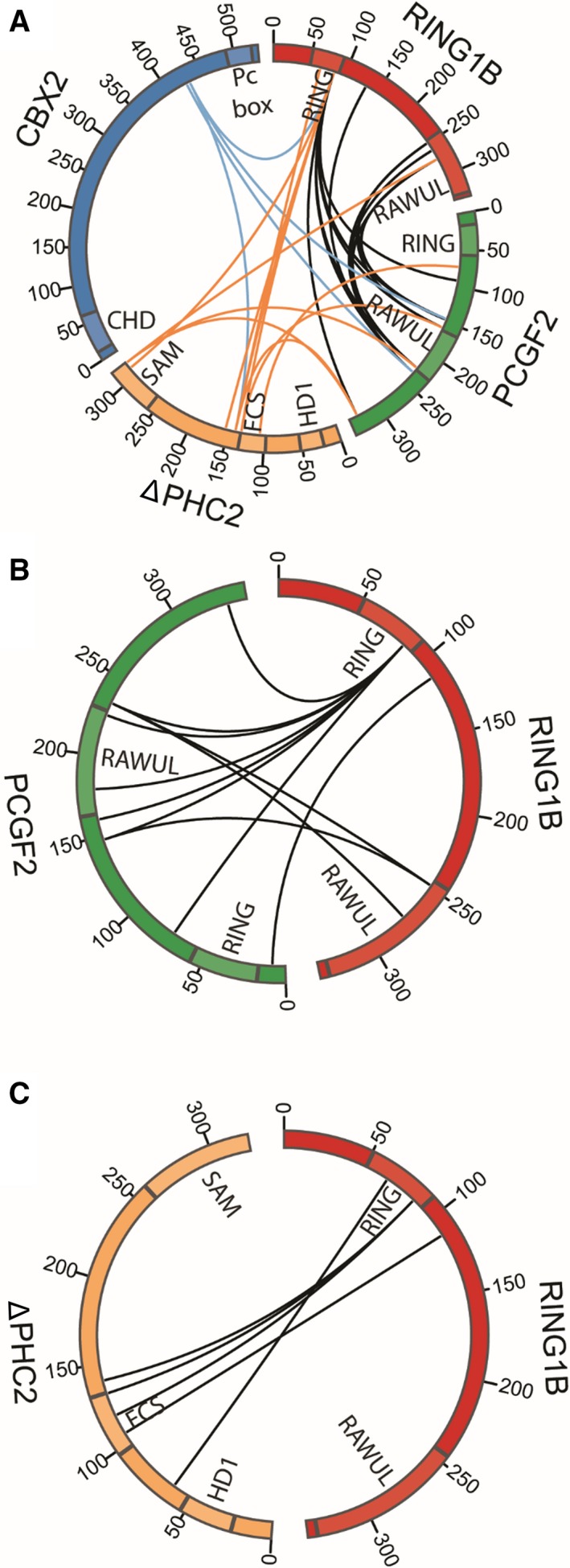
Map of the inter‐subunits interactions of ΔPRC1.2 (A), RING1B‐PCGF2 (B) and RING1B‐ΔPHC2 (C) identified by crosslinking mass spectrometry. The black lines indicate the crosslinked peptides between RING1B and PCGF2. The blue lines represent the crosslinked peptides between CBX2 and the other subunits. The orange lines represent the crosslinked peptides between ΔPHC2 and RING1B and PCGF2. Images were created with circos.

Having established that CBX2 and ΔPHC2 are important for compaction of ΔPRC1.2, we mapped the inter‐subunits interactions of ΔPRC1.2 by crosslinking mass spectrometry. We used DSS as a crosslinker agent for lysine residues at a C_α_‐C_α_ maximum distance of 27 Å. Our data recapitulate inter‐subunit interactions known from available crystal structures of PRC1 domains 11, 13, 14. For instance, our data show that RING1B and PCGF2 interact through their RING and RAWUL domains. Interestingly, we could also identify novel interactions in regions for which no crystal structure is currently available. For instance, the FCS zinc finger domain of ΔPHC2 interacts with the RING domain of RING1B and the SAM domain of ΔPHC2 interacts with the RAWUL domain of PCGF2. Moreover a region close to the Pc box in CBX2 establishes interactions with the RING domain of RING1B, the RAWUL domain of PCGF2 and a region close to the FCS zinc finger domain of ΔPHC2 (Fig. 4). We could confirm this novel interaction between the FCS domain of ΔPHC2 and the RING domain of RING1B by performing XL‐MS also on the purified RING1B‐ΔPHC2 heterodimer (Fig. 4).

To understand if any rearrangement of RING1B and PCGF2 occurs when ΔPHC2 and CBX2 are absent, we also performed crosslinking mass spectrometry on the isolated RING1B‐PCGF2 subcomplex. In the isolated heterodimer, RING1B is forming interactions with the RAWUL domain of PCGF2 similar to ΔPRC1.2 complex. In ΔPRC1.2, the RAWUL domain of PCGF2 is also interacting with ΔPHC2 (Fig. 4). This observation is in agreement with the reported structure of the RAWUL domain of PCGF4 and the partial HD1 domain of ΔPHC2 14.

**Table 3:** Identification of ΔPHC2 and RING1B in the RING1B‐ΔPHC2 subcomplex by peptide mass fingerprinting mass spectrometry.

Protein_id	top3	ssm	usm	upm	max_score	total_score	% Sequence coverage
ΔPHC2	7.376497	1630	44	44	207	4141	78.6
RING1B	7.315681	43	13	13	150	1273	39.5

**Table 4:** Summary of the AUC experiment of reconstituted nucleosomes.

	Predicted mass (kDa)	Observed mass (kDa)	Frictional ratio	Sedimentation coefficient (S)	S_20_w
Nucleosome	206	206	1.4	7.1 ± 0.2 S	11.5 ± 0.3 S

### ΔPHC2 and CBX2 subunits do not affect H2A ubiquitination on mononucleosomes

**Figure 5:**
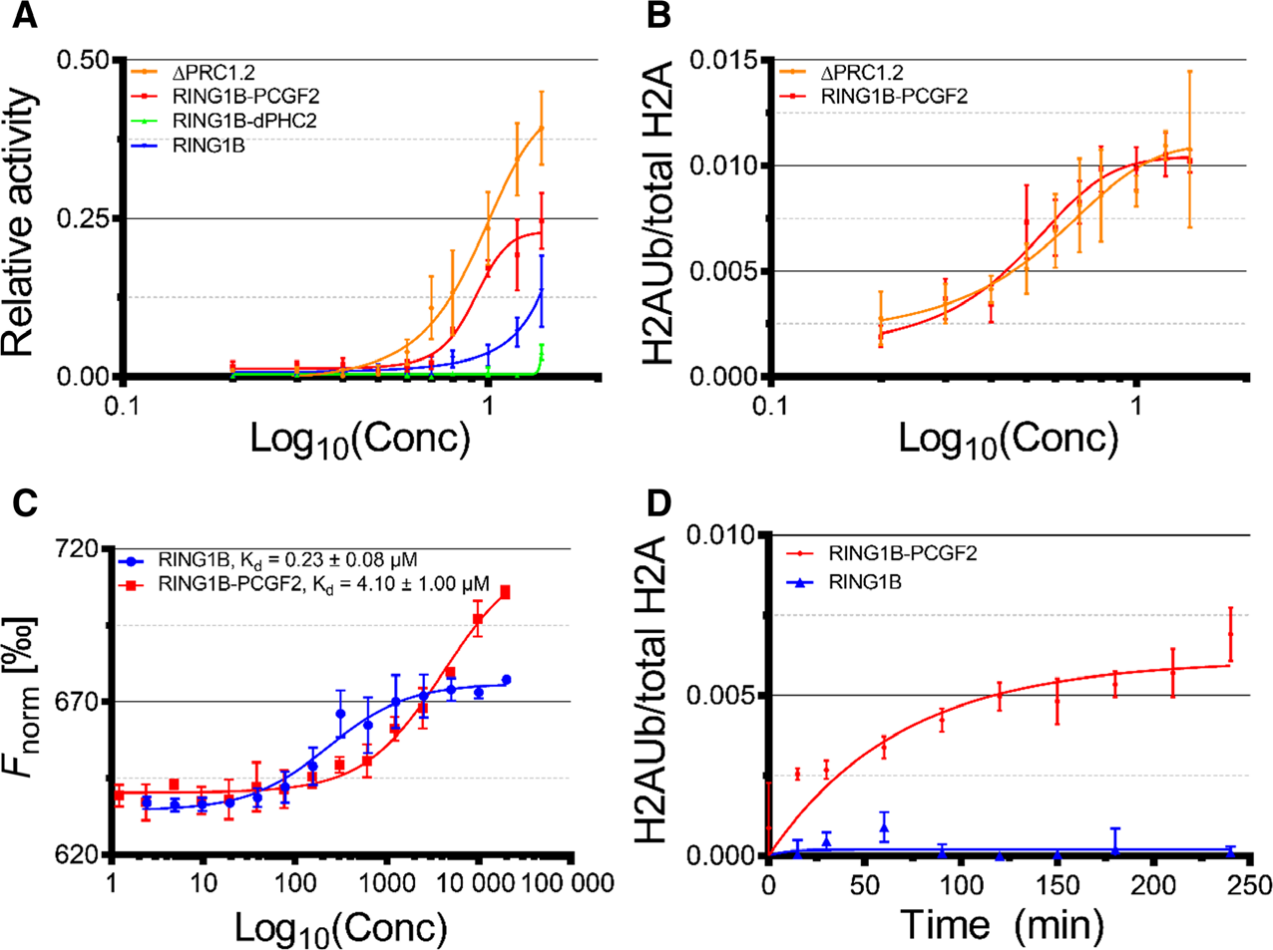
(A) Plot reporting the monoubiquitination activities of ΔPRC1.2 and its subcomplexes, as measured by western blot. (B) Plot reporting the monoubiquitination activities of ΔPRC1.2 and RING1B‐PCGF2, as measured using Cy5‐labelled nucleosomes. (C) Deconvoluted plots from microscale thermophoresis titrations of fluorescently labelled UbcH5c with RING1B and RING1B‐PCGF2. *K*_d_ were determined by data fitting in GraphPad. (D) E2‐discharging assay for RING1B and RING1B‐PCGF2. The error bars in all panels correspond to the standard error of the mean of at least *n* = 3 independent experiments.

Having established that the catalytic RING domain of RING1B interacts with or is in close proximity to motifs of ΔPHC2 and CBX2, we asked if these latter two subunits can modulate the enzymatic activity of ΔPRC1.2. To address this question, we measured the E3‐ligase activity of ΔPRC1.2 and compared it with the activity of its subcomplexes. We measured the E3 ligase activity of ΔPRC1.2 and its subcomplexes on mononucleosomes by quantification of western blot bands using specific antibodies against free histone H2A and ubiquitinated histone H2A. We observed that RING1B is poorly active, as previously reported 8. Coupling RING1B to ΔPHC2 (RING1B‐ΔPHC2 heterodimer) does not improve activity, whereas PCGF2 (RING1B‐PCGF2 heterodimer) boosts RING1B activity substantially (Fig. 5A,B, Figs S2 and S5). Finally, coupling ΔPHC2 and CBX2 to the RING1B‐PCGF2 heterodimer (ΔPRC1.2 complex) does not improve activity further, i.e. the ΔPRC1.2 complex and the RING1B‐PCGF2 heterodimer display similar activity (Fig. 5A,B, Figs S2 and S5). These data suggest that CBX2 and ΔPHC2 do not stimulate the enzymatic activity of PRC1.

### PCGF2 activates RING1B by reducing its affinity for the E2 enzyme

Having established a structural rather than functional role for CBX2 and ΔPHC2 within ΔPRC1.2, we set out to address the mechanism by which PCGF2 increases ΔPRC1.2 ubiquitination activity. PCGF2 may affect binding of RING1B to its two substrates, namely the nucleosome, as previously proposed 15, or the E2 enzyme, as previously proposed for PRC1 31, 32 and for other ubiquitin ligases such as APC/C 33. To test these hypotheses, we performed two sets of assays. First, we measured the affinity of RING1B and RING1B‐PCGF2 for UbcH5c by microscale thermophoresis (MST, Fig. 5C). We expressed and purified UbcH5c (Fig. S4), labelled it on Cys85 with the fluorescent dye NT‐547 and measured its affinity to RING1B and RING1B‐PCGF2. The *K*_d_ for RING1B‐PCGF2 is 4.1 ± 1 μm, similar to the 7 μm value reported for a minimal RING1B‐PCGF2 complex encompassing only the two RING domains 32. By contrast, RING1B exhibits a higher affinity for UbcH5c (*K*_d_ = 0.23 ± 0.08 μm). Second, we performed E2‐discharging assays to compare single‐turnover kinetic parameters of RING1B and RING1B‐PCGF2 (Fig. 5D and Fig. S6). In this assay, RING1B‐PCGF2 is active (*v*_max_ = 0.007 ± 0.001 min^−1^), whereas RING1B is inactive.

## Discussion

In this work, we report the topological mapping by XL‐MS of a canonical PRC1 isoform and of two subcomplexes, and we explore the role of the non‐catalytic subunits of this complex in regulating the biochemical and enzymatic properties of PRC1.

Our PRC1 inter‐subunit interaction map shows a novel interaction between the catalytic RING domain of RING1B and the FCS zinc finger domain of ΔPHC2, a region currently not covered by available crystal structures (Fig. 1). Our biochemical data also show that RING1B and ΔPHC2 can associate independently from the other subunits (Fig. 2). The FCS zinc finger domain of ΔPHC2 is conserved in PHC1 and PHC3, suggesting that these PHC orthologues may interact with RING1B in a similar manner as PHC2.

Additionally, our XL‐MS map of ΔPRC1.2 shows a new interaction between the RAWUL domain of PCGF2 and the SAM domain of ΔPHC2 (Fig. 4). Recently, a complex between the HD1 domain of ΔPHC2 and the RAWUL domain of PCGF4 was crystallised 14. The residues of PCGF4 interacting with ΔPHC2 are conserved in PCGF2, suggesting that such interaction is maintained between ΔPHC2 and PCGF2. The HD1 domain of ΔPHC2 is close to its FCS zinc finger domain (Fig. 1). This latter domain is interacting with the RING domain of RING1B. By combining our XL‐MS data with available crystal structures, it emerges that RING1B, ΔPHC2 and PCGF2 are spatially close to each other. Interestingly, CBX2 is less tightly connected to the rest of the complex, but it does come in close proximity to the catalytic module *via* its Pc box domain, which interacts with all the other subunits. An interaction between the Pc box of an orthologue of CBX2 (CBX7) with the RAWUL domain of RING1B had been captured previously 13. Such close proximity of ΔPHC2 and the Pc box of CBX2 to the catalytic RING1B‐PCGF2 heterodimer, along with our SAXS data showing similar dimensions for ΔPRC1.2 and RING1B‐PCGF2, suggest a structural role for the ΔPHC2 and CBX2 subunits in supporting the architectural organisation of PRC1.

By contrast, despite their important structural role, ΔPHC2 and CBX2 do not affect the H2AK119 monoubiquitination activity of RING1B‐PCGF2 (Fig. 5A,B, Figs S2 and S5). The limited impact of CBX2 on the cPRC1 activity is in agreement with previous data 25, 34, while the role of ΔPHC2 in catalysis was unknown. Moreover, it is worth noticing that vPRC1, in which the RYBP/YAF2 subunits replace CBX and PHC, have greatly enhanced catalytic activity with respect to the RING1B‐PCGF heterodimer, as demonstrated by ChIP‐seq experiments showing a correlation between high levels of H2AK119Ub in gene loci and RYBP localisation 9. The RYBP stimulation of the monoubiquitination activity of RING1B‐PCGF was observed also *in vitro* 4, 9, 25, 34.

Such lack of effect on catalysis does not necessarily mean that ΔPHC2 and CBX2 do not play any functional role in PRC1. The positively charged low complexity region of CBX2 is known to be responsible for inducing chromatin compaction *via* a non‐enzymatic mechanism 7, 12. In this respect, it is interesting to notice that such region does not interact with the other subunits of our PRC1 isoform. Moreover, it has been reported that ΔPHC2 induces clustering of cPRC1 at specific chromatin loci 10, an activity that could be carried out *via* modulation of self‐association by the SAM domain of PHC2 10, 35.

Finally, our enzymatic and biochemical data provide insights into the role played by the PCGF subunit in modulating nucleosome binding and ubiquitination activity of the PRC1 catalytic subunit RING1B. Such role of PCGF remains still largely unclear, despite the structure of the RING domains of RING1B and PCGF4 bound to mononucleosomes being solved 15. Previous reports suggest that PCGFs may enhance RING1B activity at various steps of catalysis, i.e. by modulating recognition of the substrates or inducing allosteric modulation of the RING1B active site. For instance, certain PCGF4 mutations at the nucleosome interface (i.e. R64A) cause both a 10‐fold decrease in nucleosome affinity and a 2‐fold decrease in activity. However, some mutations at the same interface (i.e. K62A) have a more limited effect on both affinity and activity (*K*_d‐K62A _= 0.37 μm *vs K*_d‐WT_ = 0.23 μm, and 80% of wild type activity preserved), while others even abolish activity but increase nucleosome affinity (i.e. E33A, *K*_d‐E33A_ = 0.09 μm) 15. Additionally, key residues far from the nucleosome interface but close to the active site, namely K73 and D77, which are conserved both in PCGF2/4 (canonical PRC1) and in PCGF1/3/5/6 (non‐canonical PRC1), ensure the correct orientation of ubiquitin for the reaction and their mutation results in a lower intrinsic E3 ligase activity 32. Yet, all these studies have been performed using minimal catalytic PRC1 modules formed by the RING domains of RING1B and PCGFs. For full‐length complexes, which are more difficult to produce in large quantities and homogeneous conformations, data is sparser. Here, we analysed two aspects of the PCGF‐RING1B interaction using full length PCGF2 and RING1B. First, by single turnover E2‐discharging assays we found that PCGF2 activates RING1B, likely by increasing its affinity to the substrate nucleosomes (Fig. 5D). Second, by microscale thermophoresis, we found that PCGF2 decreases the affinity of RING1B to the E2 enzyme [*K*_d (RING1B) _= 0.23 ± 0.08 μm,* K*_d (RING1B‐PCGF2) _= 4.1 ± 1.0 μm], probably preventing that a too tight RING1B‐E2 interaction inhibits catalytic turnover (Fig. 5C). At the molecular level, this difference in affinity could possibly be explained with the fact that in RING1B, the E2 enzyme may interact with residues outside the RING domain (i.e. the C‐terminal RAWUL domain), which would become inaccessible when PCGF2 binds. Independent of how the interaction actually takes place, our results, along with previous work showing that fusion of E2 to the catalytic PRC1 core increases nucleosome affinity 15, suggest that a well‐regulated interplay between the catalytic module of PRC1 and the two substrates, E2 and nucleosomes, are essential to regulate catalysis.

In summary, our analysis of the canonical PRC1 isoform ΔPRC1.2 provides a topological map of this important chromatin remodelling complex, revealing novel interactions between regions of currently unavailable high‐resolution 3D structures, and it suggests specific structural and functional roles for the non‐catalytic subunits PCGF, CBX and PHC.

## Author contributions

MM designed the study; MC and OP performed the experiments; MC, OP and MM acquired and analysed the data; MM and MC wrote the manuscript; MM obtained funding and supervised the research.

## Supporting information


**Fig. S1.** SDS/PAGE and biochemical characterisation of PRC1.2 (A) and PRC1.4 (B).
**Fig. S2.** Representative western blot membranes used for quantification of the H2A monoubiquitination activity of ΔPRC1.2 (A) and its subcomplexes (B–D) (quantification reported in Fig. 5C).
**Fig. S3.** (A) Representative native PAGE (6% acrylamide) of nucleosome core particles (NCP) stained with SYBR safe (left) and Instant blue (right).
**Fig. S4.** Purification of the UbcH5c E2 enzyme.
**Fig. S5.** Representative SDS/PAGE gels used for quantification of the H2A monoubiquitination activity of ΔPRC1.2 (A) and RING1B‐PCGF2 (B) using Cy5‐labelled nucleosomes (quantification reported in Fig. 5B).
**Fig. S6.** E2‐discharging assays.
