# Supplementary material for: Topology and enzymatic properties of a canonical Polycomb repressive complex 1 isoform
Source: FEBS Lett. 2019 May 29;593(14):1837–48. doi: 10.1002/1873-3468.13442 (PMC6771824; doi:10.1002/1873-3468.13442)

SUPPORTING INFORMATION FILE FOR

Topology and enzymatic properties of a canonical PRC1 isoform

Matteo Colombo[1], Ombeline Pessey[1], and Marco Marcia[1 *]

[1]European Molecular Biology Laboratory, Grenoble Outstation, 71 Avenue des Martyrs, Grenoble 38042, France

[*]**Corresponding author**: Marco Marcia, European Molecular Biology Laboratory, Grenoble Outstation, 71 Avenue des Martyrs, Grenoble 38042, France, Tel: +33 04 76 20 7759, E-Mail: mmarcia@embl.fr

This file contains:

- Figures S1-S6

Figure S1. SDS-PAGE and biochemical characterization of PRC1.2 **(A)** and PRC1.4 **(B).** Both complexes contain all four expected subunits after strep-tag affinity chromatography. All subunits were identified by peptide mass fingerprinting mass spectrometry (here shown for PHC2 only, with matched peptides indicated by red circles and corresponding m/Z values listed on the right). CBX2, PCGF2, PCGF4, and RING1B were additionally identified by Western blot (WB).

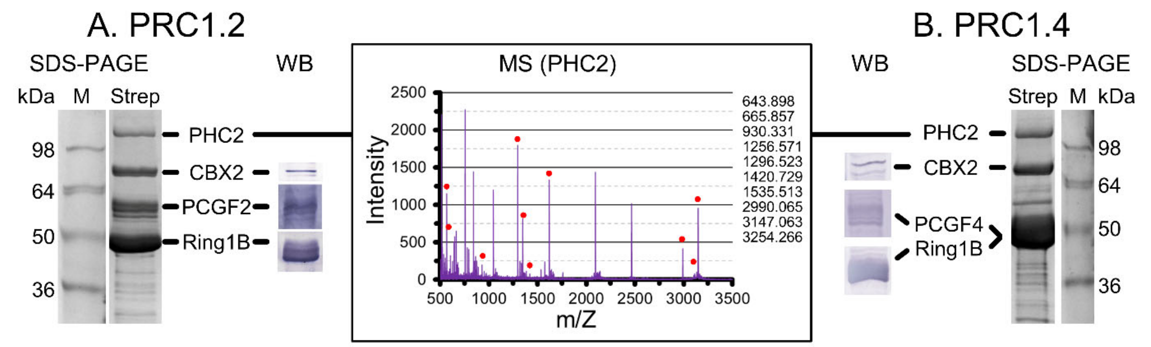

Figure S2. Representative Western blot membranes used for quantification of the H2A monoubiquitination activity of ΔPRC1.2 **(A)** and its subcomplexes **(B-D)** (quantification reported in **Figure 5C**). The orange arrow indicates the free H2A histone (detected with an antibody coupled to Alexa fluor 488 nm) while the purple arrow indicates ubiquitinated H2A (detected with an antibody coupled to Alexa fluor 532 nm).

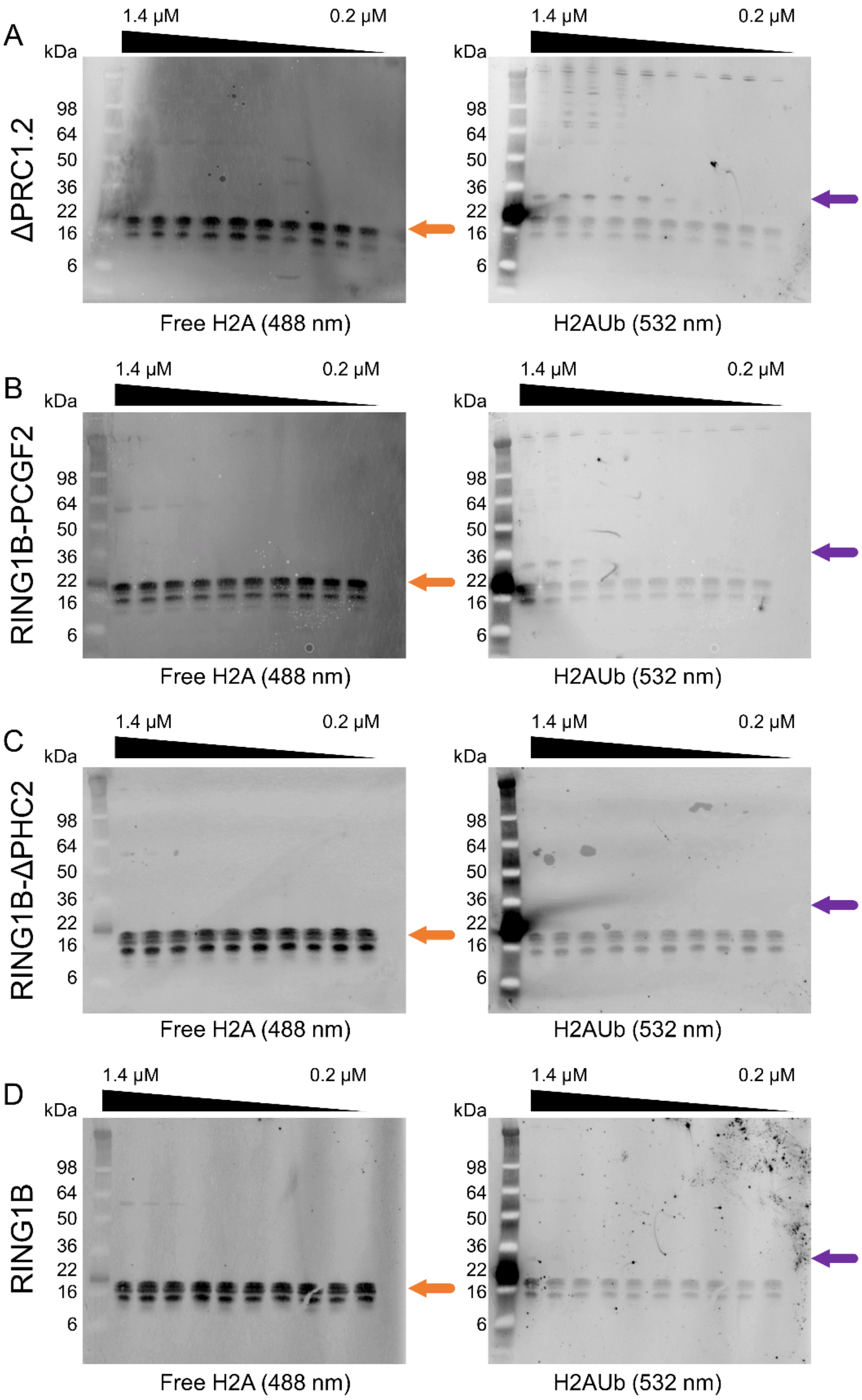

Figure S3. A) Representative native PAGE (6 % acrylamide) of nucleosome core particles (NCP) stained with SYBR safe (left) and Instant blue (right).

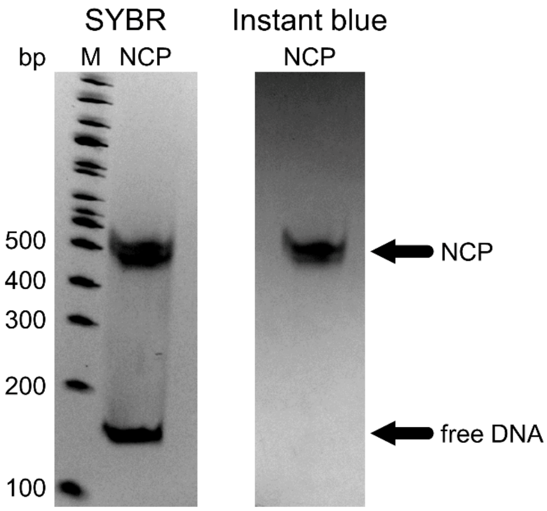

Figure S4. Purification of the UbcH5c E2 enzyme. **A)** Representative SEC profile. The orange rectangle indicates the fractions of purified E2 used in this work. **B)** Representative SDS-PAGE gels showing the different steps of purification. M is a molecular size marker, W is the wash and E the elution fraction of the first GST-affinity chromatographic step, 3C indicates the digestion product of 3C precission protease, FT is the flow through of a Ni-NTA column used to remove undigested, His- and GST-tagged E2, and SEC is the eluate from the gel filtration column shown in panel A (orange rectangle). The black arrow on the right indicates the band corresponding to the E2 enzyme.

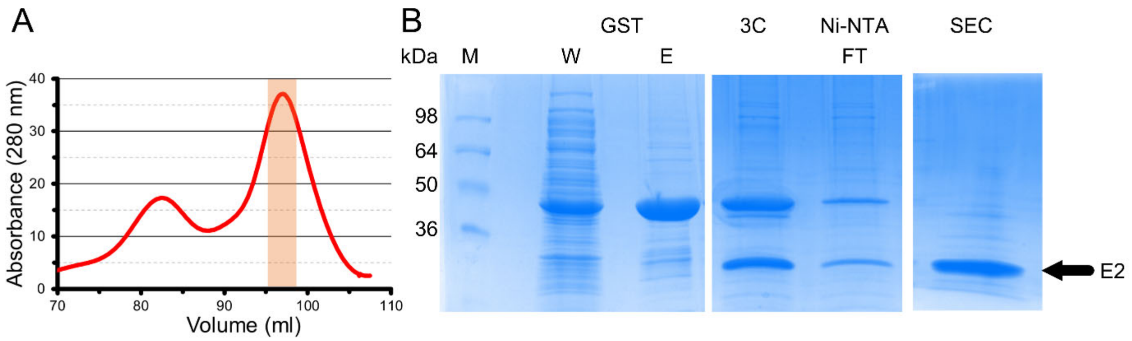

Figure S5. Representative SDS-PAGE gels used for quantification of the H2A monoubiquitination activity of ΔPRC1.2 **(A)** and RING1B-PCGF2 **(B)** using Cy5-labelled nucleosomes (quantification reported in **Figure 5B**). The orange arrow indicates the free H2A, while the purple arrow indicates ubiquitinated H2A.

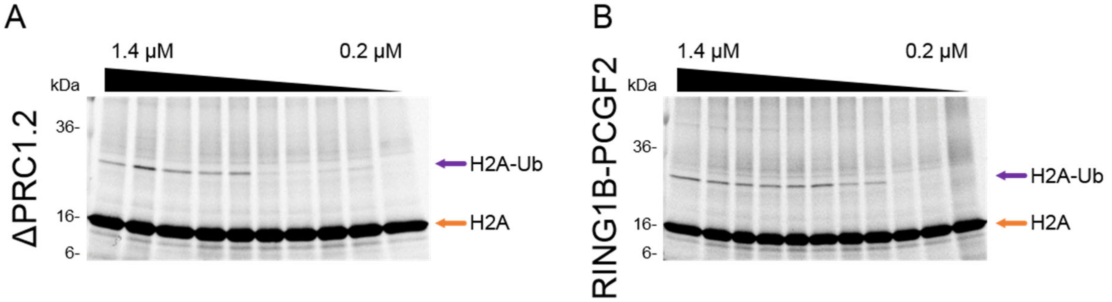

Figure S6. E2-discharging assays. **(A)** Representative SDS-PAGE gel documenting ubiquitin preloading on UbcH5c before apyrase treatment. Arrows on the right of the gel indicate free ubiquitin (Ub), free UbcH5c (E2) and ubiquitinated UbcH5c (E2-Ub), respectively. **(B)** Representative SDS-PAGE gels used for quantification of E2-discharging activity of RING1B (top) and RING1B-PCGF2 (bottom; quantification reported in **Figure 5D**). Cy5-labelled free and ubiquitinated H2A are indicated by the orange and purple arrows, respectively.

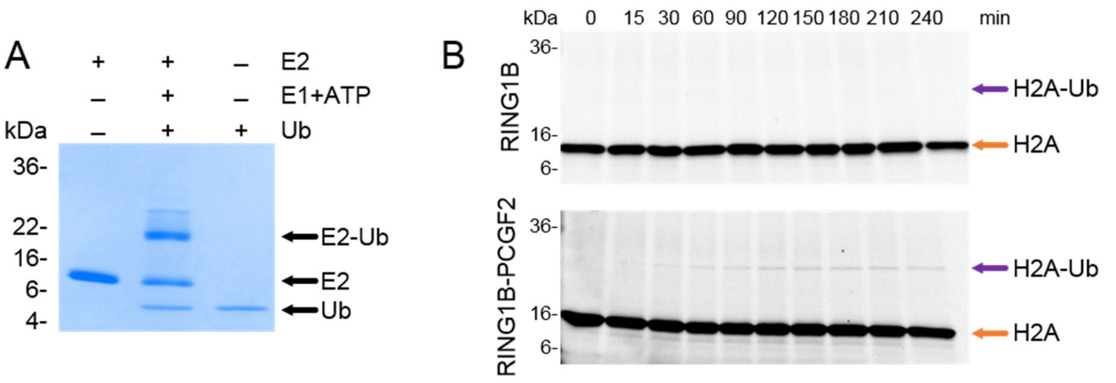

Supplement: Supplementary file 1 — Fig. S1. SDS/PAGE and biochemical characterisation of PRC1.2 (A) and PRC1.4 (B). Fig. S2. Representative western blot membranes used for quantification of the H2A monoubiquitination activity of ΔPRC1.2 (A) and its subcomplexes (B–D) (quantification reported in Fig. 5C). Fig. S3. (A) Representative native PAGE (6% acrylamide) of nucleosome core particles (NCP) stained with SYBR safe (left) and Instant blue (right). Fig. S4. Purification of the UbcH5c E2 enzyme. Fig. S5. Representative SDS/PAGE gels used for quantification of the H2A monoubiquitination activity of ΔPRC1.2 (A) and RING1B‐PCGF2 (B) using Cy5‐labelled nucleosomes (quantification reported in Fig. 5B). Fig. S6. E2‐discharging assays. [file FEB2-593-1837-s001.pdf]
